# Visual Object Recognition with 3D-Aware Features in KITTI Urban Scenes

**DOI:** 10.3390/s150409228

**Published:** 2015-04-20

**Authors:** J. Javier Yebes, Luis M. Bergasa, Miguel Ángel García-Garrido

**Affiliations:** Department of Electronics, University of Alcalá, Alcalá de Henares 28871, Spain; E-Mails: bergasa@depeca.uah.es (L.M.B.), garrido@depeca.uah.es (M.Á.G.-G)

**Keywords:** 3D-aware features, object recognition, KITTI, DPM, stereo-vision

## Abstract

Driver assistance systems and autonomous robotics rely on the deployment of several sensors for environment perception. Compared to LiDAR systems, the inexpensive vision sensors can capture the 3D scene as perceived by a driver in terms of appearance and depth cues. Indeed, providing 3D image understanding capabilities to vehicles is an essential target in order to infer scene semantics in urban environments. One of the challenges that arises from the navigation task in naturalistic urban scenarios is the detection of road participants (e.g., cyclists, pedestrians and vehicles). In this regard, this paper tackles the detection and orientation estimation of cars, pedestrians and cyclists, employing the challenging and naturalistic KITTI images. This work proposes 3D-aware features computed from stereo color images in order to capture the appearance and depth peculiarities of the objects in road scenes. The successful part-based object detector, known as DPM, is extended to learn richer models from the 2.5D data (color and disparity), while also carrying out a detailed analysis of the training pipeline. A large set of experiments evaluate the proposals, and the best performing approach is ranked on the KITTI website. Indeed, this is the first work that reports results with stereo data for the KITTI object challenge, achieving increased detection ratios for the classes car and cyclist compared to a baseline DPM.

## 1. Introduction

For environment perception, the basic human sensory sources while driving are the eyes and brain training, *i.e.*, the visual perception of the scene and the previous knowledge about traffic rules. Typically, drivers can be assisted by intelligent systems, like GPS devices and onboard visual and audio alerts coming from processing units of ultrasonic and radar signals. Indeed, the current evolution from advanced driver assistance systems (ADAS) to driverless vehicles is pursuing the integration of smarter systems that can provide more autonomy to road vehicles, which are becoming robotic platforms with several installed sensors. Among them, vision sensors grant a higher level of abstraction and semantic information that is more natural to interpret by humans compared to other sensing modalities [1]. Although they are currently employed in the automotive industry for advanced assistance functionalities [2,3], providing image understanding to road vehicles is a key issue that the research community and the industry have been recently working on and will continue doing [1,4,5].

To that end, it must be noted that man-made environments are very dynamic, *i.e.*, moving vehicles, pedestrians, cyclists, urban structure and road changes. Therefore, providing a 3D scene understanding from images requires learning descriptive models from large datasets and inferring objects and scene layout, among others. In the particular case of object detection from images, the DPM part-based detector [6] has been successfully tested on image classification, segmentation and retrieval tasks [7] during the last few years. However, inferring the location and orientation of objects for autonomous robotic platforms is still an open problem [8]. Indeed, there is a strong research interest in the object classes ‘car’, ‘pedestrian’ and ‘cyclist’ [9] to achieve more accurate 2D/3D predictions in complex, dynamic and naturalistic urban scenarios. Thus, considering the benefits of pictorial structures and the mixture of models, this paper employs DPM as a baseline and extends it for the KITTI urban scene understanding challenge [10].

**Figure 1:**
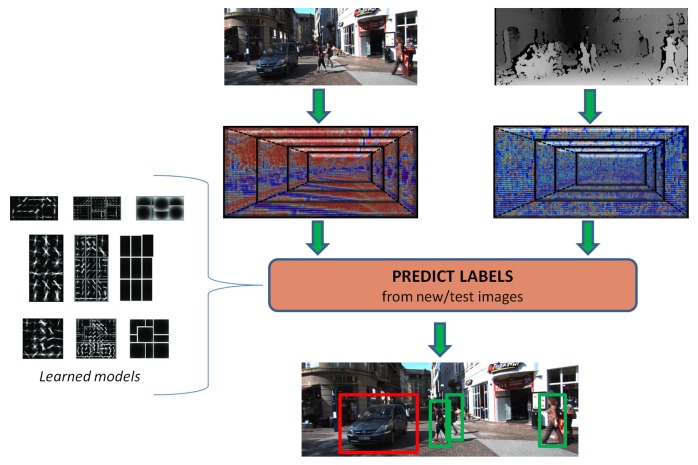
Predicting roads participants from 2.5D data. 3D-aware features from scale pyramids are computed on color and disparity images and incorporated into DPM [6].

The focus of this research work is on predicting relevant object instances contained in road scenes while employing stereo data, *i.e.*, color and disparity, as illustrated in Figure 1. In particular, we revisit the discriminatively-trained part-based models (DPM) [6]. We extend the DPM training pipeline to account for depth and color information, which is in the form of a set of proposed 3D-aware features. In addition, a set of cross-validated experiments show the performance of our proposals against a baseline DPM, and our best performing approach is publicly ranked on the KITTI website for assessing the detection ratios on the test set.

In the remainder of the paper, we start with the main related works. Afterwards, Section 3 analyzes 3D point clouds from objects in KITTI scenes and introduces the 3D-aware features. Then, the DPM is briefly reviewed and its extension to integrating the 3D-aware features is presented in Section 4. This is followed by the Experimental Section 5, and finally, Section 6 provides final remarks and future work.

## 2. State-of-the-Art

The DARPA Urban Challenge [4] was a breakthrough for autonomous vehicles, in which the competitors based their systems on manually-labeled maps, aerial imagery, GPS signals, radar sensors and accurate and expensive LiDAR devices. Vision cameras and image processing techniques were almost not employed for real obstacle detection and environment perception. These approaches demonstrated autonomous navigation on highways and non-naturalistic pathways (lacking realistic numbers of vehicles, obstacles, pedestrians and urban structure). However, urban scenarios remain a big challenge due to their naturalistic complexity, GPS signal loss and the need for very accurate maps, which are also impractical.

Therefore, to provide image understanding capabilities to autonomous vehicles, some of the urban challenges may include: object detection under occlusion [11], estimation of object orientation on 3D scenes [12], detection at far distances [13], determining the geometric layout of the scene [14], dealing with varying illumination conditions [15], appropriate modeling and parametric learning of complex scenes [16] and the generation of naturalistic datasets.

Particularly, this paper competes in the object detection and orientation estimation challenge released by the recent KITTI Vision Benchmark Suite [9]. In this challenge, excluding modified Bag of Words (mBoW) [17] and Fusion-DPM [18], which rely on laser data, the remaining proposals are based on visual appearance from color [11,19–21]. Moreover, several entries propose a modification on top of DPM [6] and have reported results for only one of the classes. Similarly, most of the works have only published performances for the object detection without considering the joint location and viewpoint estimation. Compared to them, our work is the first approach based on stereo data that has been evaluated on the KITTI object benchmark. Furthermore, we provide results for all of the categories and also for the joint object detection and orientation estimation.

### 2.1. Object Detection and DPM

Object detection from images has been under active research since the 1970s and is closely related to the beginning of computer vision a decade before. Actually, the early work on pictorial structures and spring-like object parts [22] has motivated the flourishing of several approaches for visual recognition of objects [7]. For example, BoF (Bag of Features) approaches [23] were fruitful multi-class categorization algorithms applied to natural scenes and intelligent vehicles. However, they had the shortcoming of not predicting the object location, which was solved with multiple kernels [24] also employing visual words. In contrast, [25] announced Exemplar-SVMs, which are compositional models trained from only one positive example and millions of negative ones.

Additionally, pedestrian detection has received a lot of attention in the last few years to reduce road fatalities [26]. Despite the advances to this extent, the generalization towards recognizing wider sets of road participants in complex, dynamic and naturalistic urban scenarios [8] has motivated the application of DPM and its variants. In [27], a brief review of related works is provided, some of them tested in KITTI [11,21] or in other image sets [28,29]. Basically, these works made some adaptations of the DPM training pipeline and the underlying mixture of models to account for intraclass variability, occlusions and objects' viewpoints.

### 2.2. 3D Reasoning

Extending DPM to account for the 3D peculiarities of the objects can leverage the semantic scene understanding. In [12], the object location and viewpoint estimation was proposed as a structured prediction, and the models were learned from 3D geometric constraints with the support of synthetic CAD models. On the other hand, more complex methods have devised a higher level of abstraction, *i.e.*, to include a 3D cuboid model [30], in which DPM is extended in the feature and filter size to learn objects 3D location and orientation from monocular images. Alternatively, in [31], a joint object detection and occlusion reasoning approach is formulated as a novel structured Hough voting scheme for indoors and extracting visual features from RGB-D data.

In this sense, Section 3.1 introduces a discussion about the problem of recognizing road participants with 3D point clouds recovered from stereo images.

### 2.3. Color and Disparity: Features and Approaches

DPM uses HOG descriptors computed from color images to learn the appearance patterns of objects. These features have been widely employed since the seminal paper [32] in conjunction with a linear SVM classifier. After this, 3DHOG (3D Histograms of Oriented Gradients) [33] was devised as a spatio-temporal descriptor based on HOG and the extraction of interest points with the Harris3D detector, applying the algorithm to action recognition in videos. Similarly, we extend the gradient features for 3D-awareness, but in our case looking for an object description without temporal meaning.

In contrast, [34] combined color and disparity cues in the HOS (HOG computed on stereo) and DispStat (disparity statistics) features for pedestrian detection. The first one was based on the HOF-like feature [35] extracted from the depth field, but in the case of HOS, it was directly computed on the disparity map. The second one (DispStat) was based on a simple average of disparity values in a HOG cell. Their experiments showed a lower miss rate when concatenating several descriptors (HOG + HOF + HOS + DispStat), which suggests the benefits of adding depth/disparity cues for the pedestrian detection problem. Our work confirms this hypothesis with our proposed 3D-aware features and extending the analysis to cars and cyclists of the larger KITTI dataset.

Furthermore, our work explores the open discussion on which approach would be the best one: better features and more data or better models and learning algorithms [16,36]. Some recent works have also explored the limits of the HOG feature space, proposing the novel histograms of sparse codes (HSC) that are directly learned from data [37] or devising a feature pooling [38], which relaxes the fixed cell grid of the original HOG to learn more representative features from color and gradients.

## 3. Visual and Depth Description of Objects in 3D Urban Scenes Reconstructed from Stereo-Vision

### 3.1. Problem Description

Let us consider a moving observer with a stereo camera on it, which is navigating through structured and non-open spaces, such as streets inside cities or interurban roads. In this context, we are interested in the 2D detection and viewpoint estimation of cars, pedestrians and cyclists, which is one of the tasks posed by the KITTI Vision Benchmark Suite [10]. This suite provides a dataset collected in urban and interurban naturalistic environments employing an autonomous driving platform. It includes stereo images, positioning data and dense 3D point clouds from LiDAR. Although the Velodyne point clouds could provide an enhanced 3D scene understanding in conjunction with appearance information, it is a more expensive solution for integration in autonomous vehicles. Thus, our work studies the visual recognition of road participants when employing images from cost-effective stereo cameras.

The stereo images from KITTI have been randomly picked from several video sequences. They are provided rectified and divided into training and testing subsets. Typically, the captured scenes include occlusion and background clutter, different object viewpoints, changes in scale, truncation, varying illumination conditions, shadows and color differences.

**Figure 2:**
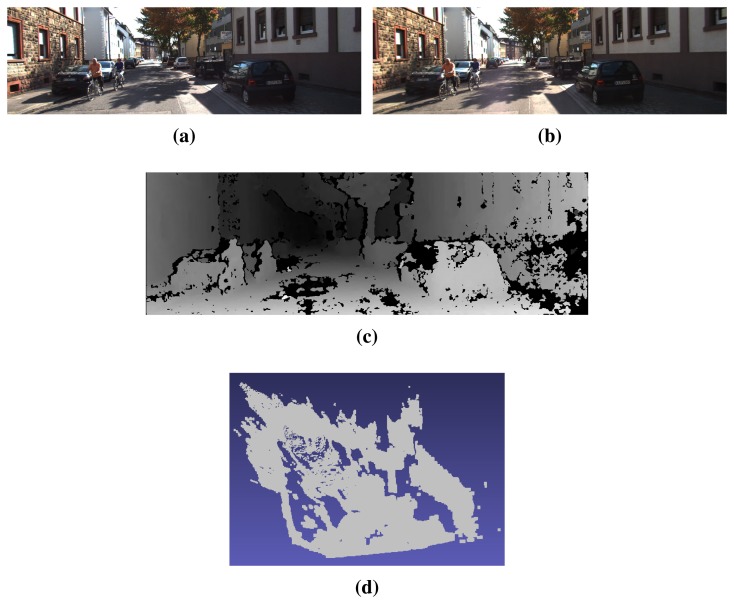
3D reconstruction of a sample urban scene from KITTI. (**a**) Left camera image; (**b**) right camera image; (**c**) disparity map; (**d**) 3D point cloud.

Our goal is to add 3D cues from the stereo images to deal with these challenges and intra-class variability and to improve the detection ratios reported by the DPM framework with monocular images [6,10,21]. Firstly, the disparity maps are required, but they are not provided in KITTI. They are estimated in our work from each pair of left-right images based on the well-known Semi-Global Matching (SGM) method [39], which provides good average performance according to the ranking in the stereo benchmark [10]. On the one hand, the addition of 3D cues could be approached from the 3D reconstructed scene. Figure 2d represents the recovered point cloud given the calibration parameters provided in KITTI. Intuitively, only some parts of the scene structure and some perspective projection effects can be visually perceived. On the other hand, cars and cyclists can be clearly identified in Figure 2c.

**Figure 3:**
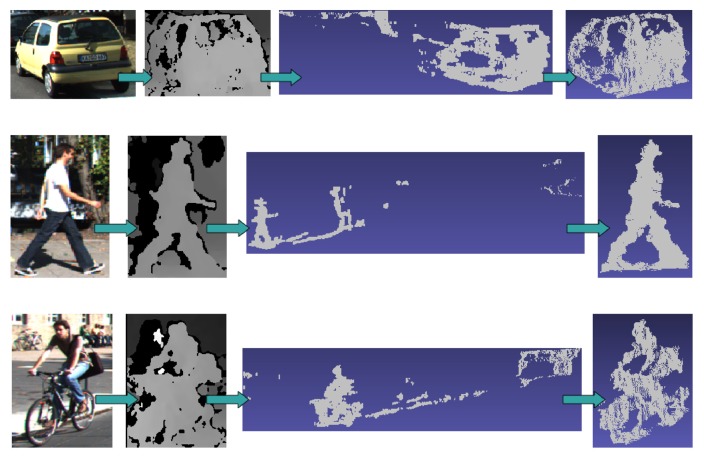
From left to right: Ground-truth color image patches, disparity patches in gray scale, original point clouds recovered from them and manually segmented objects. The point clouds are directly obtained with the reprojected pixels of the object bounding box. Then, the objects are manually segmented (last column). As can be seen, the 3D reconstructed scene before manually filtering contains large depth deviations associated with small errors in disparity. Therefore, collecting a clean 3D training dataset cannot be carried out by simply reprojecting the image pixels of the ground truth boxes or filtering by distance to the camera.

Carrying out 3D reasoning directly on the point clouds from these sparse images is a complex task. Searching for a 3D cuboid that surrounds every object requires a computationally-demanding learning and inference processes plus the addition of assumptions and tight constraints. Some approaches have faced this with monocular images [30], based on CAD prior models [40] or using dense laser data [17]. To evaluate the feasibility of extracting 3D object instances from the point clouds recovered from stereo, we have carried out a manual removal of outliers from the clouds in some of the training images. We were able to obtain recognizable 3D objects (Figure 3) for the closer and most contrasted instances.

**Figure 4:**
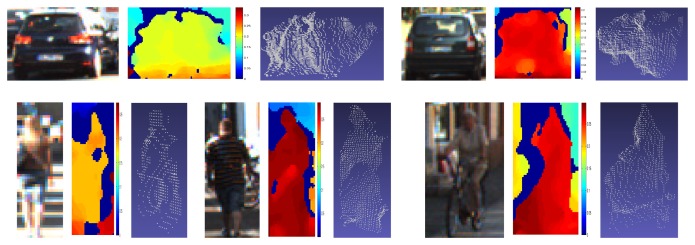
Examples of sparse point clouds manually segmented from the noisy clouds recovered from disparity. Each instance is shown in three representations: the color image patch, the disparity in a color scale and the reprojected 3D point cloud.

However, the vast majority of the dataset comprises noisy samples, as the ones depicted in Figure 4. This is due to the sparsity and small errors from disparity, which causes large depth estimations $\left(Z_{d}\propto \frac{1}{D}\right)$. Hence, automatically picking the 3D points corresponding to the 2D bounding box ground truth adds many noisy 3D points, as is demonstrated on the unfiltered point clouds in Figure 3.

Consequently, these 3D point clouds add more noise. Thus, disparity is preferred, because it carries the same information about objects, the errors do not generate large deviations and the gradients can be discriminative features. Our main aim is to learn better and richer models employing 2.5D data. Some works have also analyzed the employment of disparity in visual recognition tasks [34,41], but not in the context of DPM.

### 3.2. 3D-Aware Features

**Figure 5:**
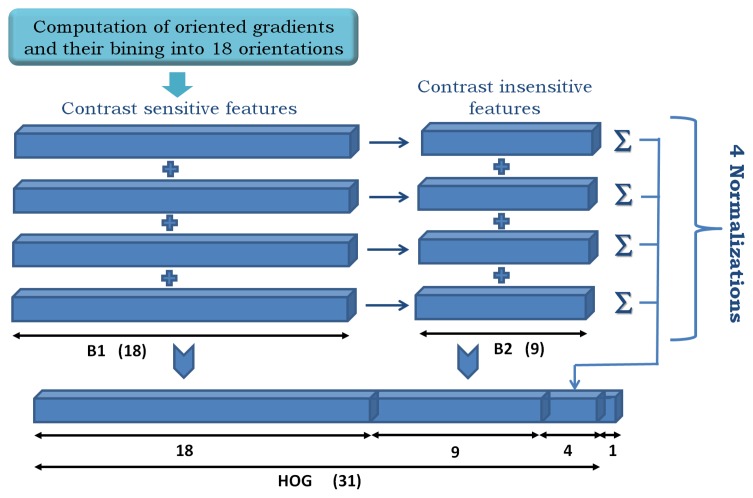
Modified HOG (Histogram of Oriented Gradients) [6]. The histograms are discretized into 18 orientations [0, 2*π*] and then normalized with four rules [32]. Afterwards, they are collapsed to the range [0, *π*], and finally, four accumulators are concatenated to form a descriptor of 31 float values, which is truncated by one for memory alignment.

Our starting point is the modified HOG features in [6], which are built as shown in Figure 5.

Given an input image patch of size *M* × *N*, it is divided into squared HOG cells of 8 × 8 pixels, and a padding of one cell is set on each border. Then, an orientation histogram of *d* = 32 elements is computed on each cell. As a result, a cube of dimensions (*h_c_* × *w_c_* × *d*) describes the input image patch, where *h_c_* and *w_c_* are the height and width in the number of cells. Figure 5 illustrates the feature construction process as a concatenation of contrast-sensitive (*B1*) and -insensitive (*B2*) gradients and four different normalizations of the histogram. The “contrast-sensitiveness” regards the number of orientations for the discretization of the gradients. *B1* are 18 bins in the range [0, 2*π*], while *B2* are nine bins reduced to [0,*π*], which is obtained by folding *B1* in two halves and adding up its elements.

These features are enhanced with 2.5D measurements in the form of color and disparity gradients, such that richer models are learned from the visual and depth appearances of the objects and additional measurement data are available for scoring bounding box hypotheses (see Section 4). Although the disparity maps are not provided in [10], we compute them from each pair of left-right images employing the SGM [39] method. Indeed, we have observed in our experiments that the gradient information from disparity maps can obtain prediction ratios close to those ones produced using gradients on color images. Consequently, the semantic information contained in the scenes is preserved in the disparity images, and discriminative models can be trained on the 2.5D data for improving the detection performance.

**Figure 6:**
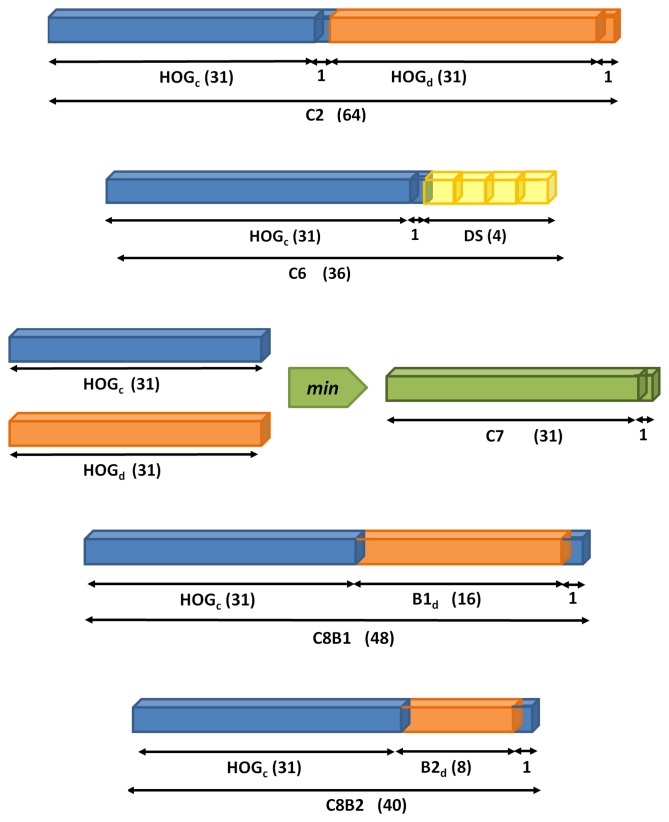
3D-aware features based on HOG and computed from 2.5D data. Subindex *c* refers to color and *d* to disparity.

Among different 3D-aware features studied, the proposed ones are in Figure 6 and described below.

**Figure 7:**
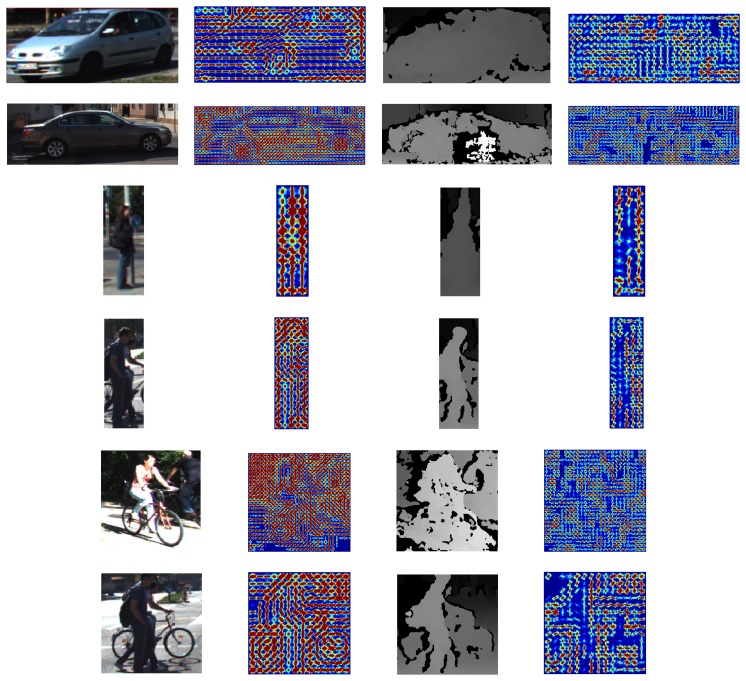
Object instances and their 3D-aware features, in particular *C8B1*. These cars, pedestrians and cyclists have been detected on the test images using our trained models. In the figure, the first two columns display the original image and the color gradients; the next two columns show the disparity patch and its gradients. The gradient features are plotted as small glyphs of the positive weights of the descriptors on a color scale. Color gradients can be visually recognized more easily. However, disparity gradients provide complementary information about the objects' depth, which leads to an enhanced description of them.

- ***C2***. Concatenation of the descriptor in Figure 5 from color (*HOG_c_*) and disparity (*HOG_d_*).
- ***C6***. The color feature *HOG_c_* is followed by four statistics (*DS*) computed on the disparity map. In particular, we propose the max, min, mean and median of disparity intensities on every HOG cell.
- ***C7***. Intersection between *HOG_c_* and *HOG_d_* computed with the element-wise minimum operation.
- ***C8B1***. The last two features focus the analysis on the importance of contrast-sensitive (*B1*) *vs.* contrast-insensitive (*B2*) histograms on the disparity. For *C8B1*, the histograms are discretized into 16 bins, instead of 18, for memory alignment purposes during the convolution of an image with the learned filters. Figure 7 depicts a set of object instances and the proposed *C8B1* features.
- ***C8B2***. Based on *C8B1* but considering contrast-insensitive features (eight bins).

## 4. Object Recognition Based on DPM

In terms of performance, pictorial structures were demonstrated in practice with DPM [6]. Besides, many of the published works in [10] rely on modifications on top of it. However, they have not exploited the use of 2.5D data, and they have not reported detailed results on different setups for its training pipeline. Thus, this work tries to fill these gaps.

**Figure 8:**
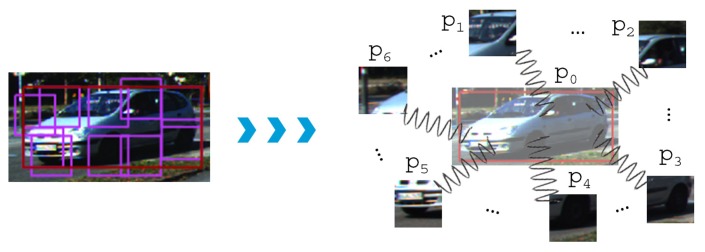
Sample pictorial representation of the spring-like connections between the root *p*_0_ (red box) and the remaining parts (*p_i_*, *i* = 1, …, 6) of the object model.

In brief, DPM classifies and locates objects at different scales based on a pyramid of modified HOG descriptors [32]. It can be viewed as a mixture of CRF (Conditional Random Fields) models, *i.e.*, one for every object viewpoint, where each of them presents a star topology as exemplified in Figure 8. Therefore, an object model consists of several parts, which are formally defined as hidden discrete random variables (*p_i_*), because they have not been annotated in the dataset. The main bounding box *p*_0_ is given by ground-truth labels, however.

Every part is defined as *p_i_* = (*u_i_*, *v_i_*, *l_i_*), which corresponds to the upper-left corner coordinates (*u*, *v*) in pixels and the scale level *l* in the feature pyramid [6]. The location, scale and size of *p*_0_ are given by the ground truth during training, but they are predicted when searching for the objects in the test images. The number of parts and their size are fixed during initialization. Besides, due to the latent nature of the parts, (*u_i_*, *v_i_*, *l_i_*) have to be estimated during both learning and inference. Mathematically, they are determined by exploring the Latent-SVM (LSVM) formulation [6] in Equation (1).

(1) $${\text{score}}_{\theta }\left(p_{0}\right)=\underset{z\in Z(\text{x})}{\max}\theta^{T}\psi \left(\text{x},z\right)$$

where **x** denotes an input image patch, ***θ*** is the parameter vector and *ψ* is the potential with the visual and deformation features of hypothesis *z* = (*p*_0_, …, *p_n_*), and their product resembles an energy function. From the set of possible configurations of object parts *Z*(**x**), the selected hypothesis *z* = (*p*_0_, *p̂*_1_, …, *p̂_n_*), exhibiting the maximum energy, provides the score for a 2D patch (*p*_0_) in the image.

Considering only one mixture component and our features, we propose to add 3D-aware parameters and potentials that capture the appearance and depth variations of the objects. Thus, we extend the scoring function of DPM, as shown in Equation (2). The potential *ψ*(**x**, *z*) introduced in Equation (1) decomposes into a sum of unary and pairwise potentials, *n* being the total number of parts.

(2) $$s(z)=\sum_{i=0}^{n}\theta_{c,i}\cdot \phi_{c}({\text{x}}_{c},p_{i})+\sum_{i=0}^{n}\theta_{d,i}\cdot \phi_{d}({\text{x}}_{d},p_{i})-\sum_{i=0}^{n}\theta_{p,i}\cdot \phi_{p}(p_{0},p_{i})+\text{bias}$$

The unary terms *ϕ_c_* and *ϕ_d_* describe the color and depth appearance, respectively, of each object part. They can be seen as the concatenation of the 3D-aware features for the subwindows and pyramid scales indicated by each hypothesis *z* ∈ *Z*(**x**). Similarly, the parameter vectors ***θ****_c,i_* and ***θ****_d,i_* are the learned filters for each part and source of data. Besides, the pairwise potentials *ϕ_p_* encode the 2D distances of the parts with respect to the root *p*_0_ [6]. Then, ***θ****_p,i_* contains four learned weights (*wx*, *wy*, *wx*^2^, *wy*^2^) for every part spring.

In addition, the orientation estimation is naturally viewed as object intra-class variation. Typically, it is approached as a subcategorization process [9,19,40] in which different clusters are initialized and lead to multiple model learning. DPM has been originally conceived of as a mixture of models, such that an additional discrete random variable *c* is defined to account for the component of the mixture, *i.e.*, the object viewpoint [27]. Then, the model dimensionality is increased up to *nc* orientations, having a final parameter vector *β* = (***θ***^1^, …, ***θ****^nc^*). As a consequence, the scores for each component are obtained from Equation (3), such that *β_c_* = ***θ****^c^* and *z′* = (*c*, *p*_0_, …, *p_n_*).

(3) $$\text{score}\left(c,p_{0}\right)=\underset{p_{1},\ldots ,p_{n}}{\max}s^{\prime }\left(c,p_{0},\ldots ,p_{n}\right)=\beta_{c}^{T}\cdot \psi \left(\text{x},z^{\prime }\right)$$

We take as baseline the LSVM-MDPM (Latent SVM—Modified Deformable Part Models) approaches [10,21]. For a direct comparison with them, we also choose 16, 8 and 4 orientations for the classes car, pedestrian and cyclist; where . The higher discretization is assigned for the classes with higher a number of samples in the KITTI dataset: 28,742 cars, 4487 pedestrians and 1627 cyclists.

**Learning:** The parameter vector *β* is estimated by training an LSVM classifier and a bootstrapping strategy for data-mining hard negative samples [6]. Moreover, we propose a set of modifications in the training pipeline for the supervised learning of DPM object models [27]. We found as key aspects the cleanliness of the data samples, the root filters initialization, the proper selection of negative samples, the overlap requirement during latent search, the fixation of the viewpoint variable *c* with ground truth labels and the addition of more flexibility by defining adaptive parts (in number and size) calculated from dataset priors. In particular, we propose to establish the size of the parts depending on the minimum size from the set of root filters, which are firstly initialized by DPM depending on the size of the object samples for each subcategory. At least two parts have to fit the width of the root filter at twice the resolution (twice as defined in [6]). Then, the number of parts for each root filter is obtained from its size and the estimated minimum size.
**Inference:** For predicting the 2D bounding boxes around the objects (cars, pedestrians or cyclists) in unseen images/scenes, a feature scale pyramid is built and walked through to generate the set of hypotheses, as depicted in Figure 1. The score of every hypothesis is obtained from Equation (3) and applying the filter convolution and matching process in [6]. Then, a maximum suppression filter sorts the scores of the candidate boxes and removes the ones that do not fulfill a maximum overlap requirement, *i.e.*, 50%. This overlap value is obtained as the area of bounding boxes intersection over the area of bounding boxes union.

## 5. Evaluation on KITTI Dataset

Many details of the KITTI benchmark and statistics from ground truth labels can be found in [9,42]. In brief, the object dataset consists of 7481 training images and 7518 test images of 1240 × 375 pixels depending on the rectification process. A thorough set of annotations is provided for the training objects, while the predictions on the test samples have to be submitted for evaluation to the KITTI website.

Our models are trained with five-fold cross-validation, and special attention is paid to the evaluation protocol in terms of metrics and the algorithm [27]. True/false positives and false negatives are sorted by score (Equation (2)) for displaying two kinds of plots: precision-recall and miss rate *vs.* false positives per image (FPPI). Besides, a single value is used to represent each curve in terms of average precision (AP), average orientation similarity (AOS) [9] and log-average miss rate (LAMR) [26]. They are estimated with Equations (4)–(7).

(4) $$AP=\frac{1}{\text{Npr}}\sum_{r\in \{0,0.1,\ldots ,1\}}\underset{\tilde{r}:\tilde{r}\geq r}{\max}p(\tilde{r})$$

(5) $$\text{AOS}=\frac{1}{\text{Npr}}\sum_{r\in \{0,0.1,\ldots ,1\}}\underset{\tilde{r}:\tilde{r}\geq r}{\max}s(\tilde{r})$$

(6) $$s(r)=\frac{1}{\left|D(r)\right|}\sum_{i\in D(r)}\frac{1+\cos\Delta_{\alpha }^{\left(i\right)}}{2}\delta_{i}$$

(7) $$\text{LAMR}=\exp\left(\frac{1}{N\text{fppi}}\sum_{f\in \{10^{-2},\ldots ,1\}}\log(mr_{\text{interp}}(f))\right)$$

*Npr* is the number of sampled recall points, which is 41 in the KITTI evaluation and; *r* and *p* are the recall and precision values, respectively. *D*(*r*) corresponds to the set of all object detections at recall *r*, and $\Delta_{\alpha }^{\left(i\right)}$ is the angle difference between the predicted and ground-truth orientations for the *i*-th detection. In addition, multiple detections are penalized, such that *δ_i_* = 0 when the detection *i* has not been assigned to a ground-truth bounding box, but *δ_i_* = 1 when there exists the minimum required overlap for the object class. *N fppi* is the number of FPPI points considered (nine as in [26]), and *mr_interp_*(*f*) is the miss rate interpolated at FPPI value *f*.

### 5.1. Experiments with 3D-Aware Features

**Figure 9:**
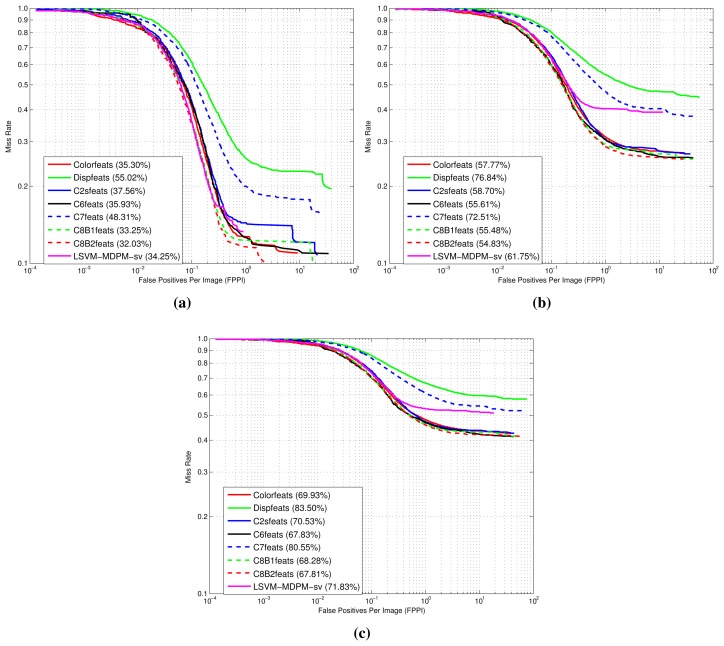
Comparison of the 3D-aware features log-average miss rate (LAMR) performance for the class ‘car’. (**a**) Easy; (**b**) moderate; (**c**) hard.

**Figure 10:**
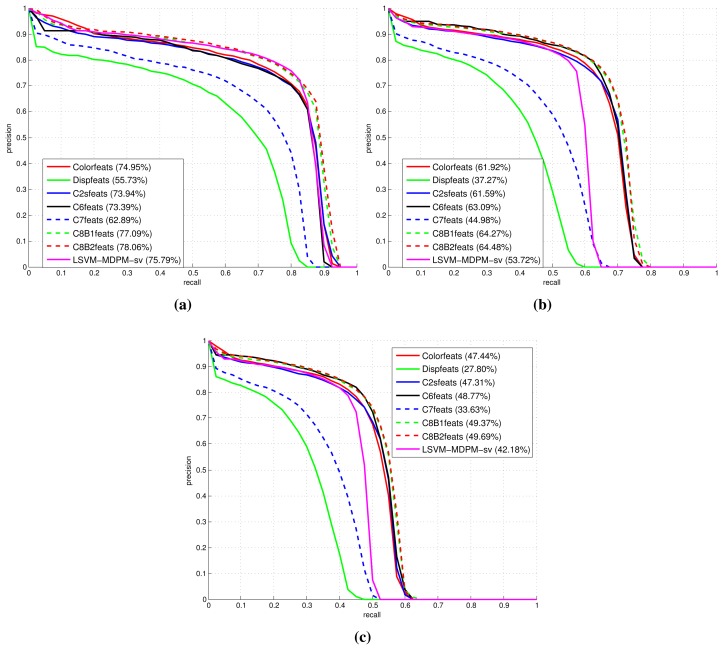
Comparison of the 3D-aware features average precision (AP) performance for the class ‘car’. (**a**) Easy; (**b**) moderate; (**c**) hard.

**Figure 11:**
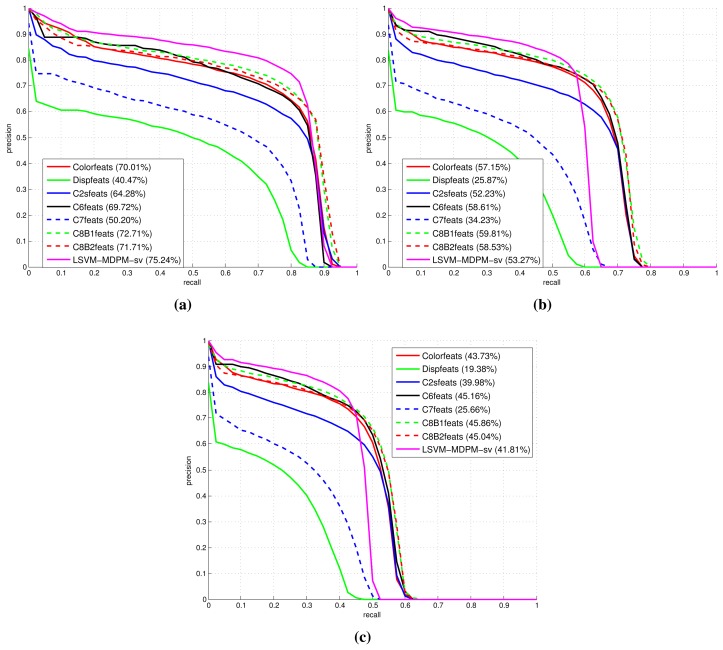
Comparison of the 3D-aware features average orientation similarity (AOS) performance for the class ‘car’. (**a**) Easy; (**b**) moderate; (**c**) hard.

Figures 9, 10 and 11 show comparative plots for cars and the 3D-aware features in Section 3.2. The curves have been averaged over the validation folds of the training subset. For supervised learning, we have employed the configuration *medium-T8* reported in [27]. Figure 9 represents miss rate *vs.* FPPI curves; Figures 10 and 11 depict precision *vs.* recall plots for detection and orientation estimation. Every figure contains three comparative graphs, which are linked to the three difficulty levels defined by the KITTI challenge [10]. Besides, they include the results for the pre-trained model LSVM-MDPM-sv (supervised training of LSVM-MDPM) that we use as a baseline.

From these validation tests, it is demonstrated that *C8B1* and *C8B2* (green and red dashed lines) yield the best object detection and orientation estimation ratios. Indeed, they present the highest AP and AOS and the lowest LAMR. Compared to *C2* (blue continuous curve), they produce a moderate boost in performance that is more clear for the viewpoint prediction. Besides, they have a shorter dimensionality than *C2* and lead to speedups, which is more notable during training, although it also benefits the prediction stage. In addition, they outperform the baseline for all difficulty levels, but the gain is more prominent for ‘moderate’ and ‘hard’ samples. Interestingly, *C8B2* (red dashed line) peaks in the AP for all evaluated levels, but *C8B1* (green dashed line) is superior for AOS, which can be explained by the nature of its contrast-sensitive features (0–2*π* gradient orientations). Indeed, *C8B1* is preferred, because it yields a 1% increase over *C8B2* at all difficulties.

The remaining tested features also produce interesting results. For example, the *C6* descriptor (black plots) presents AP, AOS and LAMR figures close to *C8B1* and *C8B2* for the difficult samples. However, it has a slight inferior performance for the ‘easy’ subset, which could be explained by the shorter length of the descriptors when more information is available (easy samples correspond to fully-visible and closer objects). In relation to *C7* (blue dashed curves), which computes the minimum between HOG color and HOG disparity descriptors, it does not show any contribution as demonstrated by its low precision values in all cases. This seems to be highly influenced by the *Dispfeats* with the lowest precision (green continuous lines) in all plots. Then, the use of disparity alone does not provide enough visual information about the objects.

In summary, the biggest gains are obtained when using the 3D-aware features *C8B1* and *C8B2*, which clearly outperform the baseline LSVM-MDPM-sv. Mainly, there are two reasons: the appropriate supervised learning of DPM [27] and the richer models learned with the employment of 2.5D data.

Furthermore, providing adaptive object parts in their number and size, which is a novel proposal compared to the seminal DPM framework, the prediction performance is further enhanced according to our cross-validation experiments for the object classes ‘car’ and ‘cyclist’. Due to paper length limitations, we only include here the detection and orientation estimation final values for the test images. They have been submitted to the KITTI website with the name *DPM-C8B1*. Tables 1 and 2 summarize the results compared to the pre-trained baseline. For a full and updated ranking, visit [10].

As can be seen from the tables, we outperform the baseline for cars and cyclists detection. The main factors contributing to this success are the appropriate supervised training, the *C8B1* features and the adaptive parts. Indeed, both false positives and false negatives are reduced because of the richer models and features from 2.5D data. However, the results for pedestrian show ratios below the baseline. This is caused in part by poor disparity measurements for distant pedestrians and false detections of cyclists, which have a very similar appearance and are usually standing at traffic lights or going on sidewalks.

In relation to runtime estimations, the average values are 28 s, 13 s and 7 s, respectively, for each category and for the joint object detection and orientation estimation. The experiments have been carried out on an i7 CPU with 4 cores @ 2.5 GHz and 16 GB of RAM. This was executed in MATLAB with some functions in C/C++ (features, stochastic gradient descent, convolution and image resizing). Excluding the recent subcategorization approach [19], we are among the state-of-the-art. Speedups can be achieved when moving the whole prediction process to optimized C++ code [43,44].

**Figure 12:**
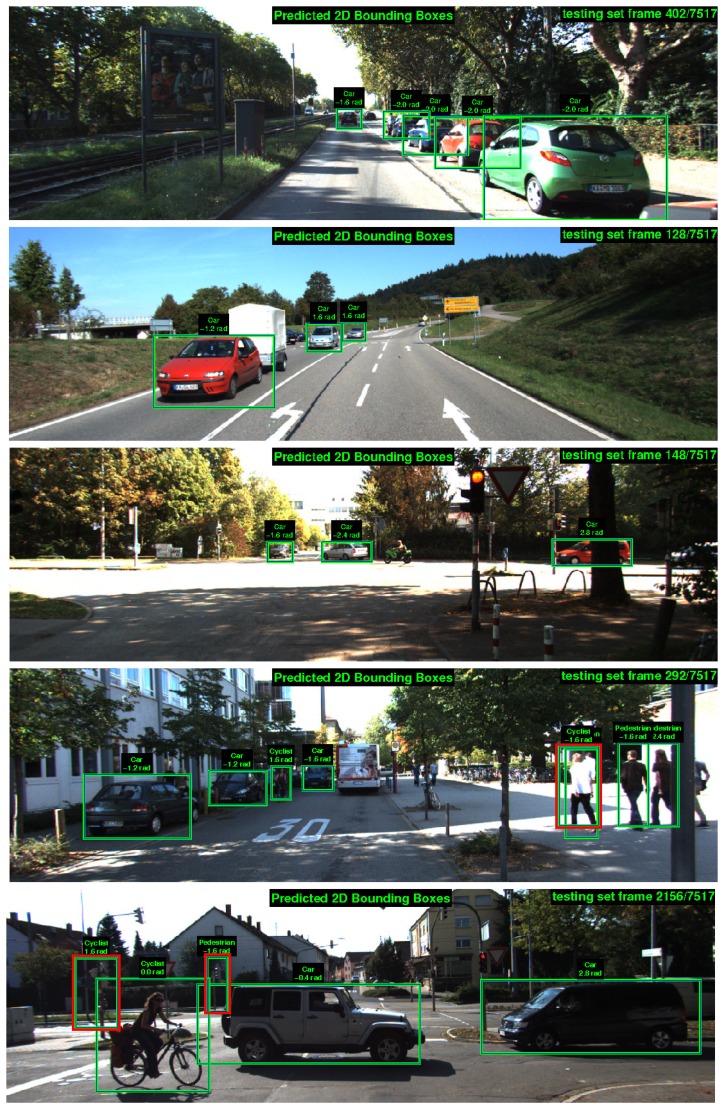
Examples of predicted labels in KITTI testing frames with true positives (green), false positives (red) and false negatives (not detected).

Figure 12 displays some prediction examples on KITTI test images. Correct detections are in green; false positives are marked in red; whilst false negatives can be identified as the cars, pedestrians and cyclists not detected in the scenes. It must be noted that trucks or vans detected as cars are neighboring classes and not counted as false positive. A similar consideration should have been done for pedestrians and cyclists, because there are many cyclists stopped at traffic lights or walking on the streets that are detected as pedestrians and *vice versa*. However, this distinction is not made by the KITTI evaluation protocol.

Although many challenging object instances are correctly detected, there are still several wrong and/or missed detections. The most typical cases are cyclists and pedestrians confused for each other, which can be interpreted as a normal detector behavior given the high similarities for some poses. Besides, there are some false positives in trees and other vertical structures of the city for these classes, which matches with the vertical gradients learned by the models. In relation to cars, there exist some miss-classifications due to background areas with similar visual appearance. The typical false negatives are the occluded vehicles in road sides where they are parked and near one another. Another source of false positives is the loose fitting of 2D bounding boxes around the objects.

## 6. Final Remarks and Future Work

This paper has presented the first research work that reports results using stereo data on the KITTI object detection and orientation estimation challenge [10]. The successful object detector, known as DPM [6], has been revisited and modified for 3D-awareness in urban scenes. The mixture of models has been extended in the number of parameters to account for features extracted from color and disparity images. As a result, the baseline LSVM-MDPM-sv has been outperformed by our approach for the classes ‘car’ and ‘cyclist’, and the results have been published on the KITTI website [10]. Besides, we have depicted the inherent difficulties in performing 3D reasoning and modeling from the sparse and noisy point clouds reconstructed from stereo images in naturalistic urban environments.

In general, prediction models have a clear target: reducing the overall error of the system when applying a trained model on new unseen data. Ideally, one would like to find the most appropriate model complexity (in terms of object parts, features and filter dimensionality, mixture components, deformation parameters, *etc.*) that minimizes both model bias and model variance. The first one is related to underfitting, while the second one to overfitting. Estimating the main source of the error in our system, bias or variance is not easy given the complexity of the DPM framework and the large intraclass variability of the KITTI objects. However, we took special care of the error measurement to favor the assessment.

Firstly, the evaluation protocol has been clearly defined, and five-fold cross-validation has been carried out for training [27]. Comparing the results from LSVM-MDPM-sv and our *DPM-C8B1* between validation and testing, we observed a relative gap between precision values that is higher for our *DPM-C8B1* approach. This could be an indication of possible high variance [45]. Increasing the number of training samples and/or reducing the features length could ideally help. In this regard, we already mirrored positive training samples [27] to double the number of samples and also tested the shorter *C8B2* features, without achieving significant changes in performance. Another typical approach to increase the data samples is jittering the geometry of the training bounding boxes around the ground-truth locations [46]. However, this is implicitly in DPM during the latent search around training objects.

In contrast, the error for pedestrians is high in the validation and testing, which is related to high bias. Therefore, we point out as possible reasons: (1) the adaptive parts, which may be causing a model complexity reduction; and (2) a poor representativeness of the features for this class that may lead to low discriminative models. In this regard, [46] already mentioned the benefits of dense stereo maps for pedestrian detection. Alternatively, [47] exploited scene geometry information to enhance a pedestrian detector. Depth cues were extracted in the form of stixels, which are directly computed from the stereo images, and they were employed to filter out detections without the need for computing disparity maps.

On the other hand, the class ‘cyclist’ yielded improved prediction for the easy samples with similar precision estimates in validation and testing, such that there is no clear symptom, bias or variance. However, we have checked that the labeled cyclists in the KITTI dataset are limited in number, and they are usually more challenging to predict.

With regard to the dilemma of “better features” or “better models”, recently, some works in top vision conferences have already stated that developing better features could help, but the designed models and learning algorithms are key factors to leverage current successful descriptors, such as HOG [16,38]. Similarly, our experiments confirm these statements when analyzing the gains of our proposals. We have observed that increasing feature dimensionality with disparity produced some precision increments depending on the object category. However, many decisions regarding the setup configuration during training [27] also had an important effect on the obtained performance. Thus, low-level details matter when one tries to outperform current state-of-the-art approaches.

Future trends point to deep learning [48], mid-level patch discovery [49] and regionlets [20] for data-mining the features and improving model learning. In addition, given the current state-of-the-art [10] in disparity map estimation, the study on how the accuracy of disparity maps affects the overall detection performance would be a new line of research. Moreover, the joint labeling of objects and the scene is a follow-up path to leverage the 3D urban scene understanding [5].

## Figures and Tables

**Table 1:** Object detection evaluation for the different classes (LSVM (Latent-SVM)).

**Category**	**Method**	**Moderate %**	**Easy %**	**Hard %**
Car	**DPM-C8B1**	**60.99**	**74.33**	**47.16**
Car	LSVM-MDPM-sv	56.48	68.02	44.18
Pedestrian	LSVM-MDPM-sv	39.36	47.74	35.95
Pedestrian	**DPM-C8B1**	**29.03**	**38.96**	**25.61**
Cyclist	**DPM-C8B1**	**29.04**	**43.49**	**26.20**
Cyclist	LSVM-MDPM-sv	27.50	35.04	26.21

**Table 2:** Joint object detection and orientation estimation.

**Category**	**Method**	**Moderate %**	**Easy %**	**Hard %**
Car	LSVM-MDPM-sv	55.77	67.27	43.59
Car	**DPM-C8B1**	**50.32**	**59.51**	**39.22**
Pedestrian	LSVM-MDPM-sv	35.49	43.58	32.42
Pedestrian	**DPM-C8B1**	**23.37**	**31.08**	**20.72**
Cyclist	LSVM-MDPM-sv	22.07	27.54	21.45
Cyclist	**DPM-C8B1**	**19.25**	**27.25**	**17.95**
